# Correction to: VULCAN integrates ChIP-seq with patient-derived co-expression networks to identify GRHL2 as a key co-regulator of ERa at enhancers in breast cancer

**DOI:** 10.1186/s13059-019-1733-0

**Published:** 2019-06-14

**Authors:** Andrew N. Holding, Federico M. Giorgi, Amanda Donnelly, Amy E. Cullen, Sankari Nagarajan, Luke A. Selth, Florian Markowetz

**Affiliations:** 1 CRUK Cambridge Institute, University of Cambridge, Robinson Way, Cambridge, CB2 0RE UK; 2 The Alan Turing Institute, 96 Euston Road, Kings Cross, London, NW1 2DB UK; 3 Department of Pharmacy and Biotechnology, University of Bologna, Via Selmi 3, Bologna, Italy; 4 Dame Roma Mitchell Cancer Research Laboratories and Freemasons Foundation Centre for Men’s Health, Adelaide Medical School, The University of Adelaide, Adelaide, SA Australia

**Correction to: Genome Biol (2019) 20:91**

**https://doi.org/10.1186/s13059-019-1698-z**

Following publication of the original article [1], the authors reported that Figs. 4 and 5 had mistakenly been transposed. Please find the correct Figs. 4 and 5 below. The original article [1] has been corrected.

**Fig. 4:**
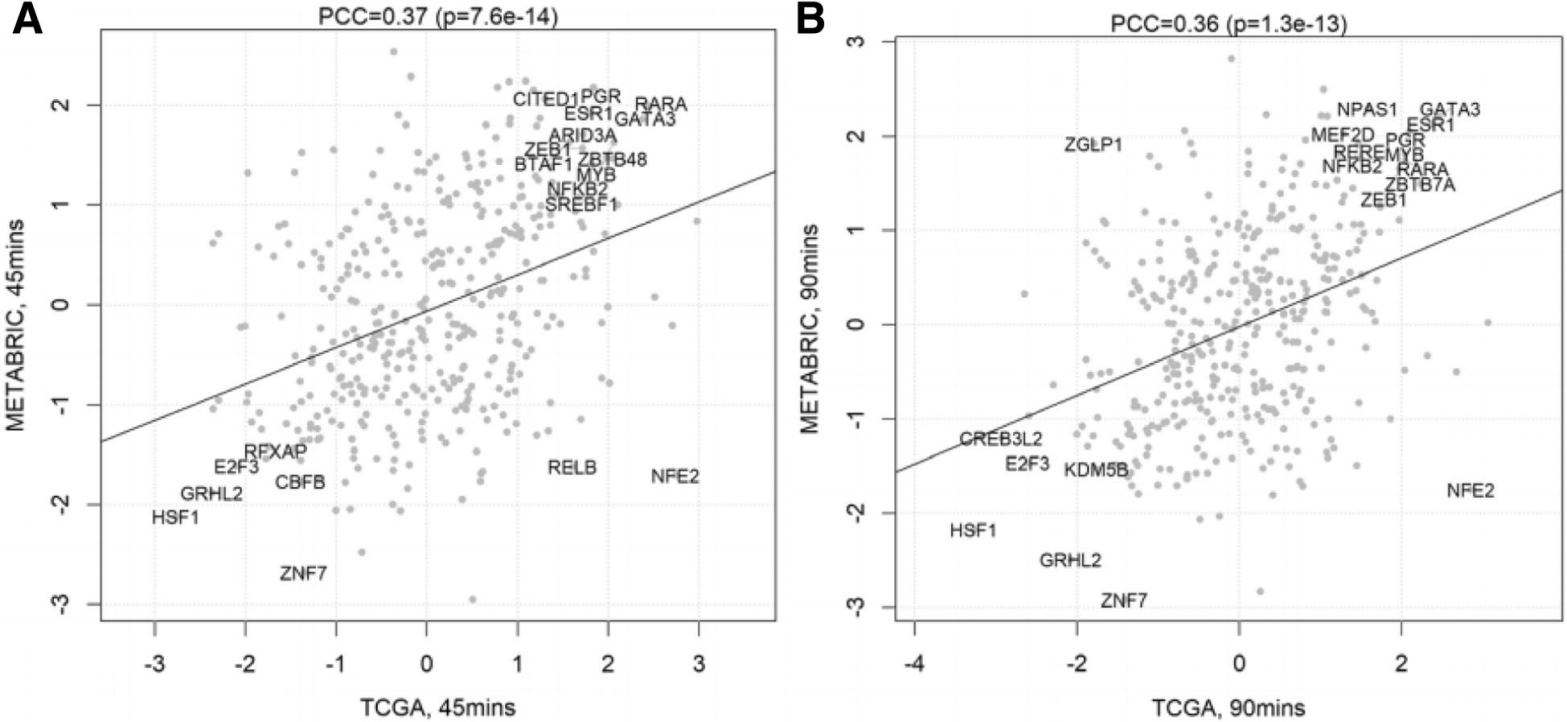
Global TF activity after estradiol treatment using different network models. XY scatter showing the TF activity as calculated by VULCAN for our differential ChIP-seq analysis of ER binding at 45 min (**a**) and at 90 min (**b**) after stimulation with 100 nM E2. Comparison of the results calculated using the METABRIC (y-axis) and TCGA (x-axis) networks shows consistent results know ER interactors including PGR, RARA, GATA3, and GRHL2. GRHL2 activity is notably enriched against. The regulon of ER is also consistently enriched in both networks. Pearson’s correlation coefficient (PCC) shown along with the significance

**Fig. 5:**
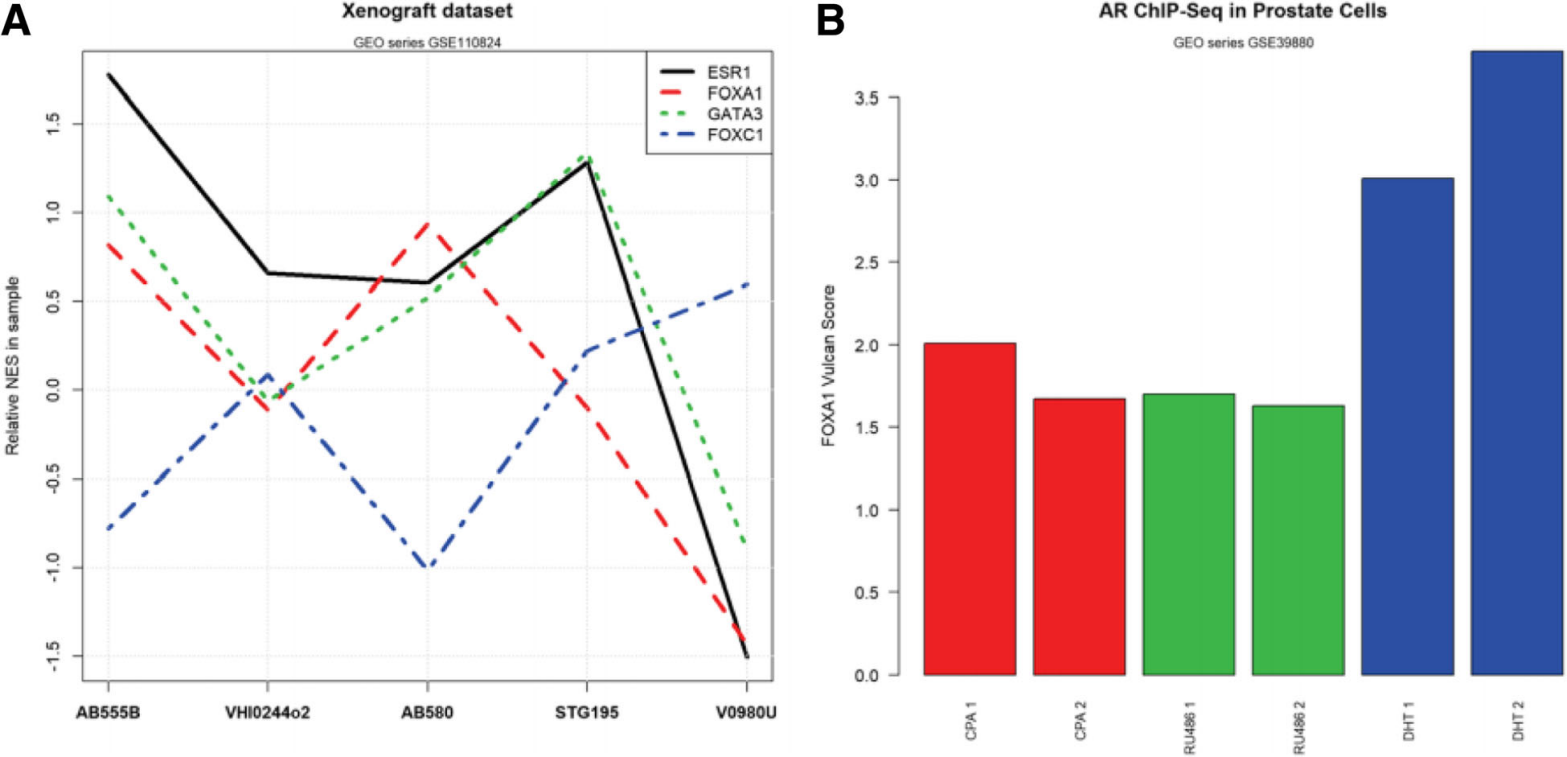
Inferring TF co-occupancy in public datasets with VULCAN. **a** VULCAN activity scores for a few TFs derived from the ER-targeted ChIP-seq breast cancer patient-derived xenograft (PDX) dataset GSE110824. The behavior of ESR1, FOXA1, and GATA3 is correlated, while FOXC1 shows an inversely correlated pattern (blue line). Interestingly, the sample with the lowest Allred score (V0980 U) has the lowest activity and the other luminal markers. **b** VULCAN activity scores for FOXA1 in ChIP-seq experiments targeting the androgen receptor (AR) in LNCaP-1F5 prostate-derived cells (dataset GSE39880). The bar plots show the relative VULCAN normalized enrichment score calculated on absolute peak intensities after treating cells with dihydrotestosterone (DHT) and partial AR modulators cyproterone acetate (CPA) and mifepristone (RU486). FOXA1 network binding is higher in the presence of the strong AR recruiter DHT. This shows an increased FOXA1/AR promoter co-occupancy in DHT-treated cells, in agreement with the conclusions of the study that originated the dataset. Two replicates for each treatment were produced and are reported in matching colors
