# Supplementary material for: VULCAN integrates ChIP-seq with patient-derived co-expression networks to identify GRHL2 as a key co-regulator of ERa at enhancers in breast cancer
Source: Genome Biol. 2019 May 13;20:91. doi: 10.1186/s13059-019-1698-z (PMC6515683; doi:10.1186/s13059-019-1698-z)
Supplement: Supplementary file 1 — Supplemental figures and tables. (PDF 3249 kb) [file 13059_2019_1698_MOESM1_ESM.pdf]

Supplementary Figures

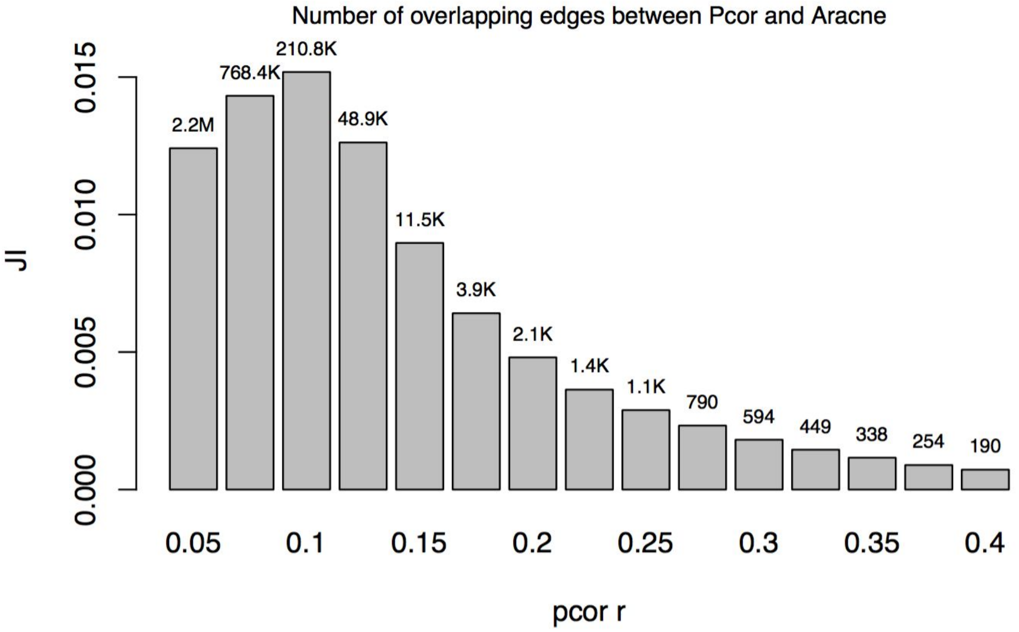

Fig. S1: Sheer number of overlapping edges at different Pcor thresholds.

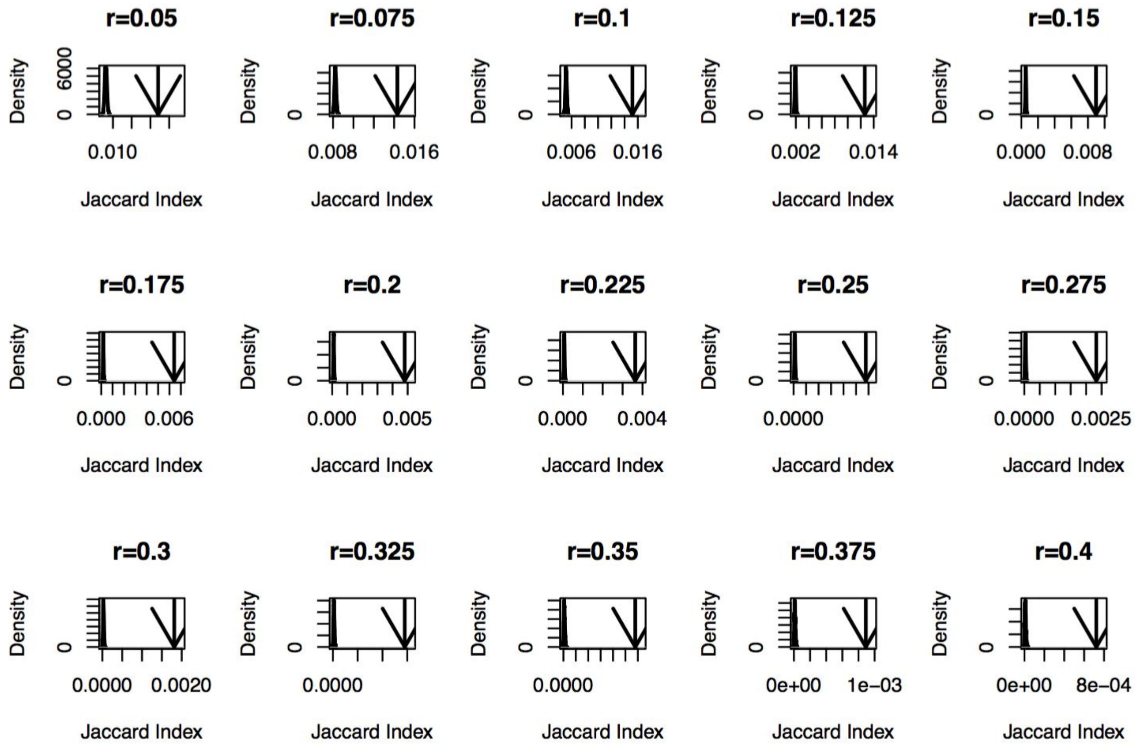

Fig. S2: Comparing the Jaccard Index between the ARACNe network and Partial Correlation networks (indicated as arrows) and Random networks (indicated as distributions).

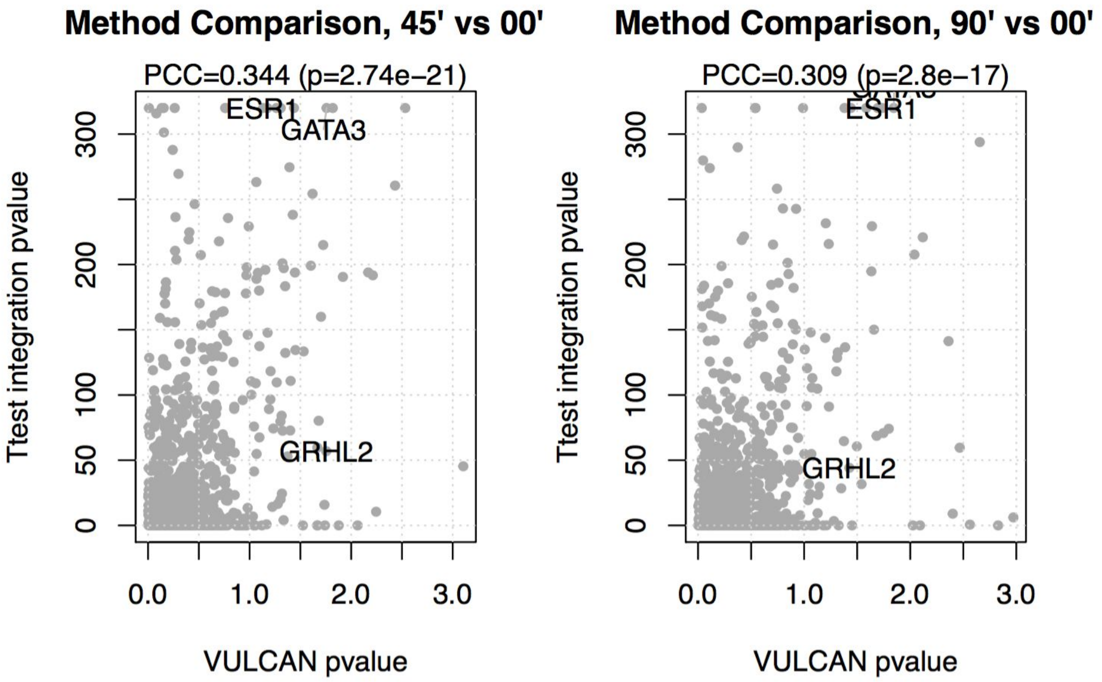

Fig. S3: **Comparison between VULCAN/VIPER and T-test integration.**

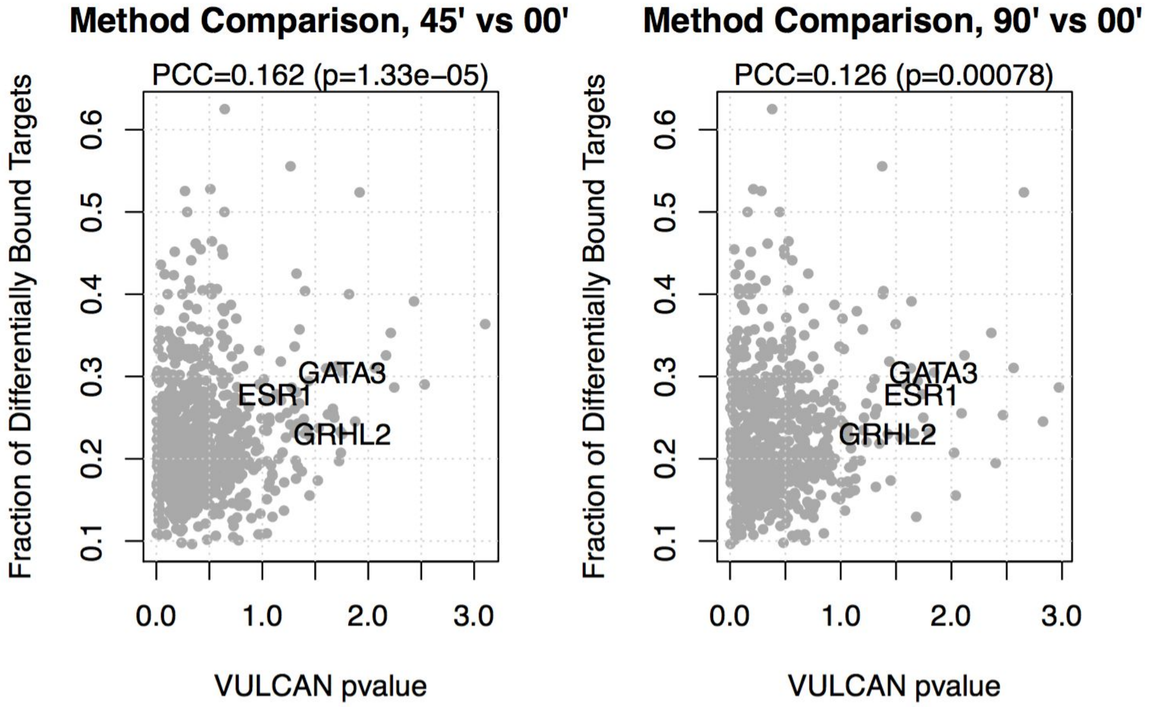

Fig. S4: Comparison between VULCAN/VIPER and a fraction of targets found method.

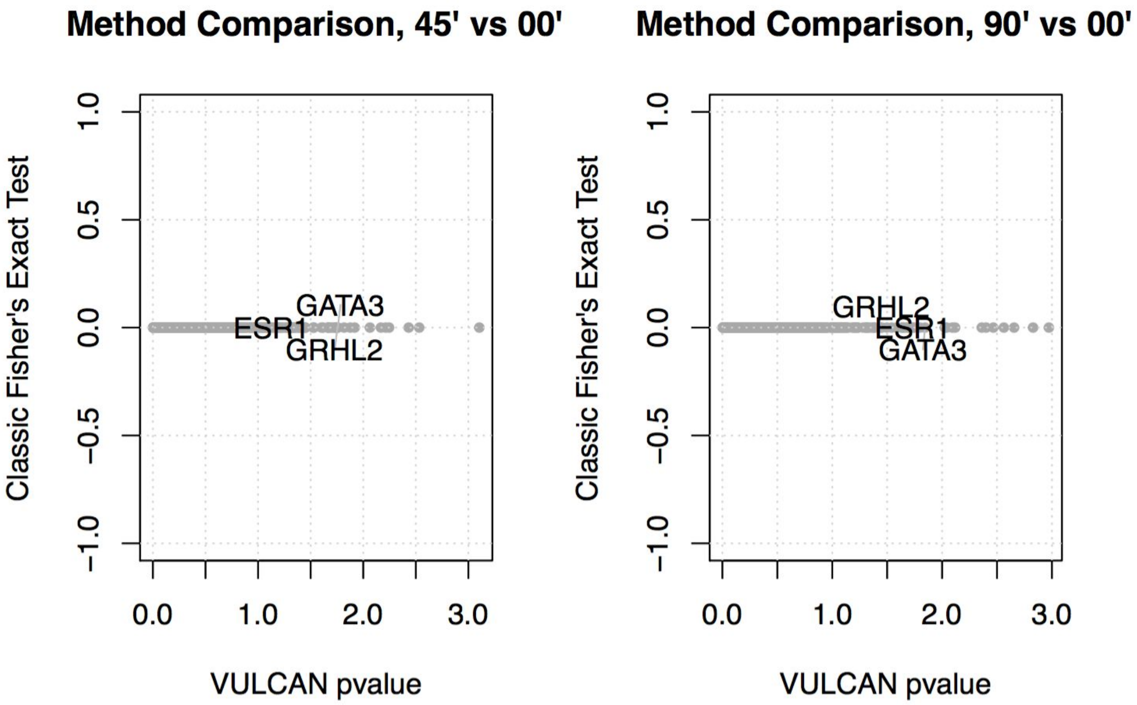

Fig. S5: Comparison between VULCAN/VIPER and Fisher's Exact Test method.

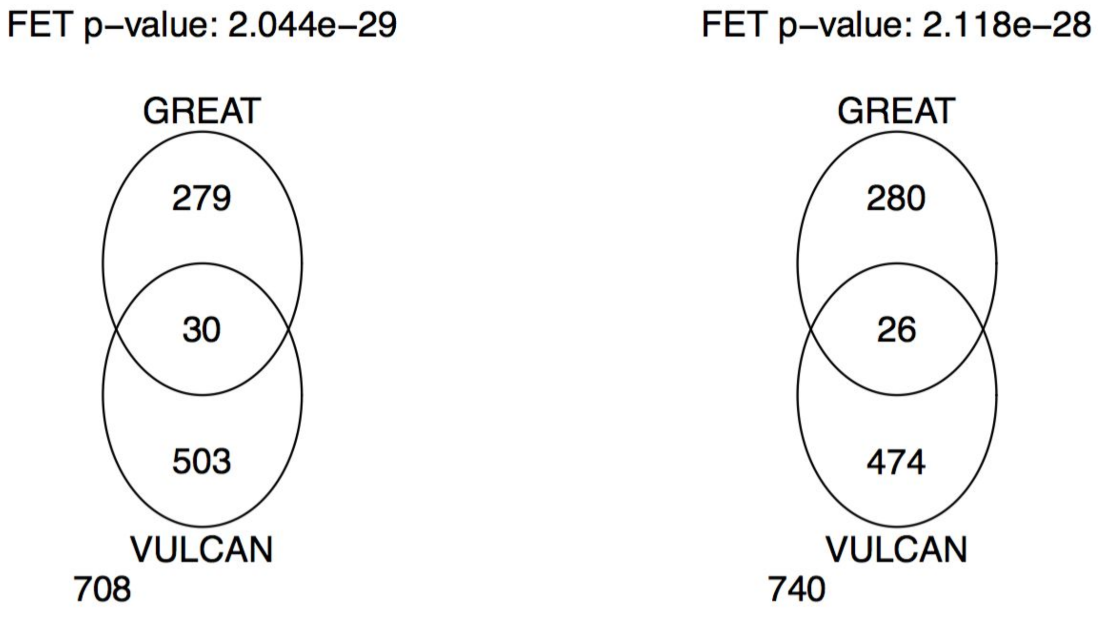

Fig. S6: Comparison of results from the VULCAN and GREAT methods.

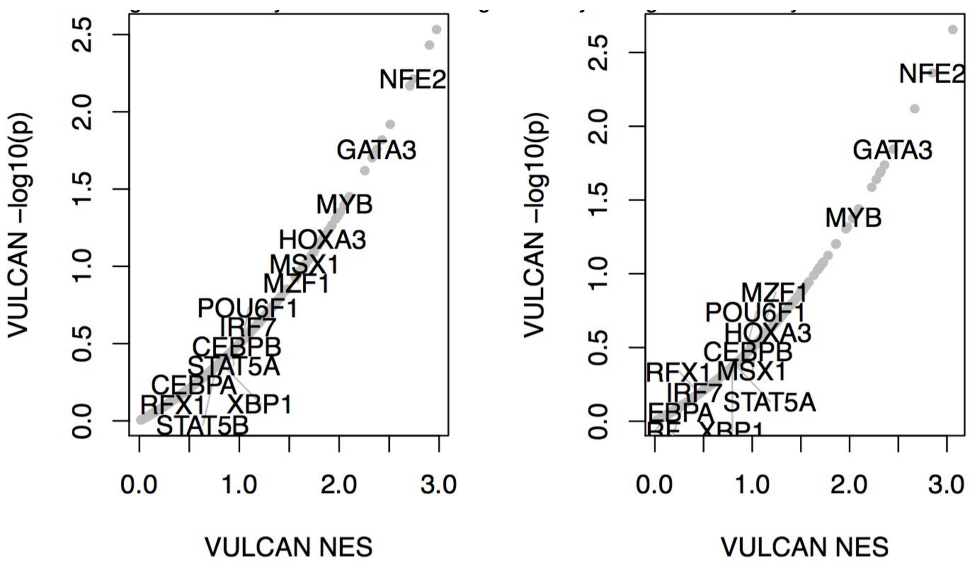

Fig. S7: Comparison of results from the VULCAN and ChIP-enrich methods.

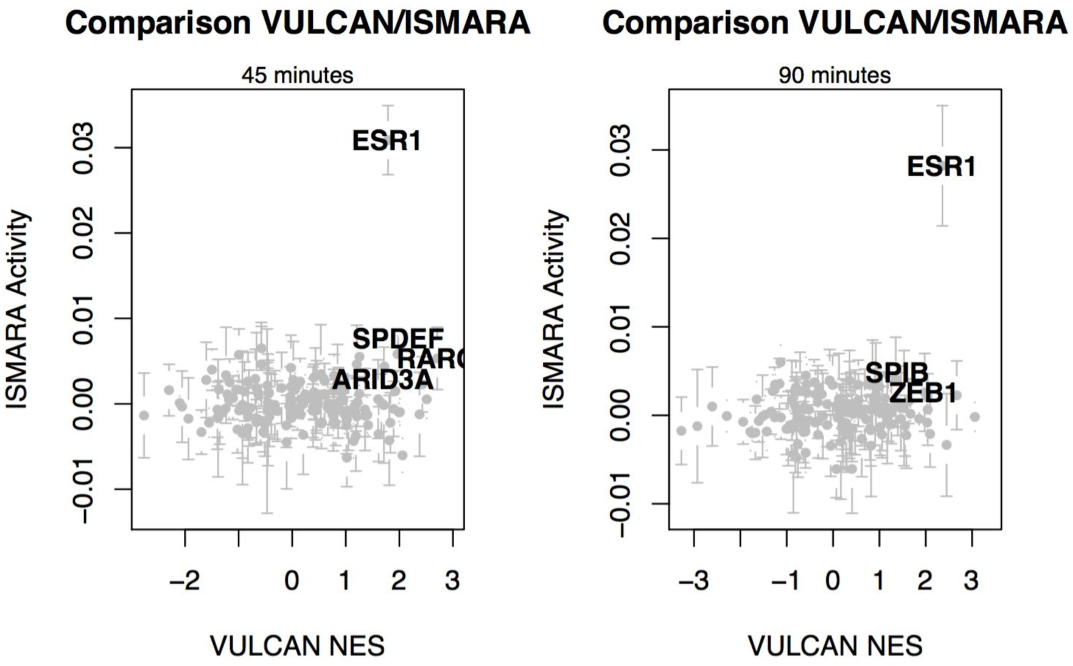

Fig. S8: Comparison of results from the VULCAN and ISMARA methods.

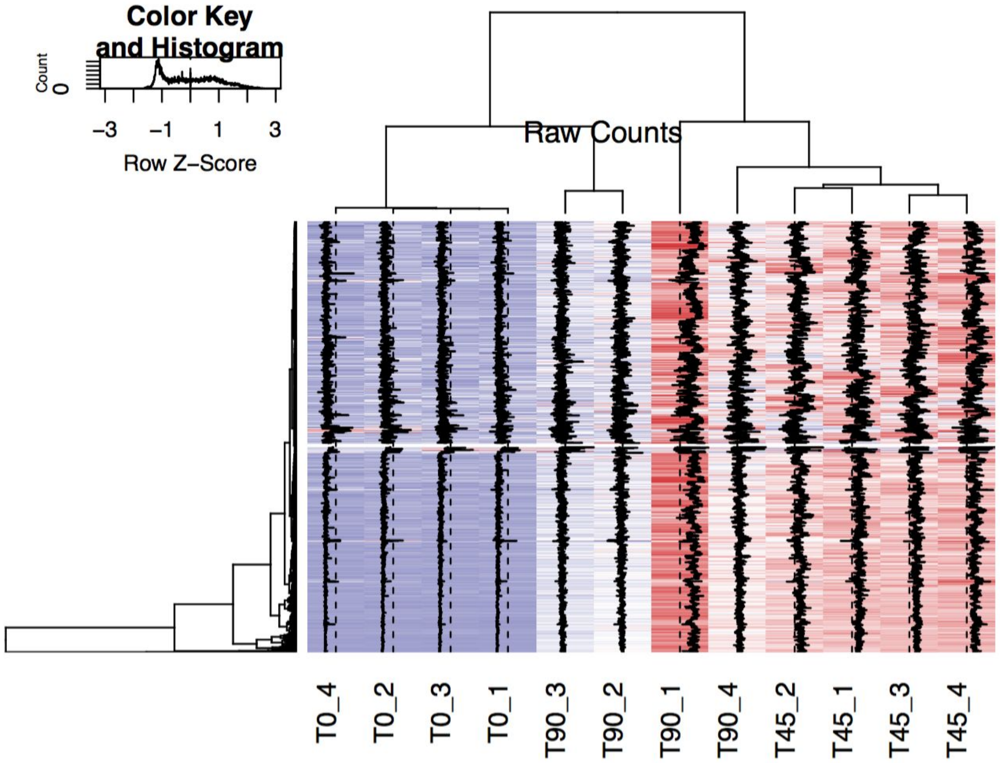

Fig. S9: Dataset clustering with Raw Counts.

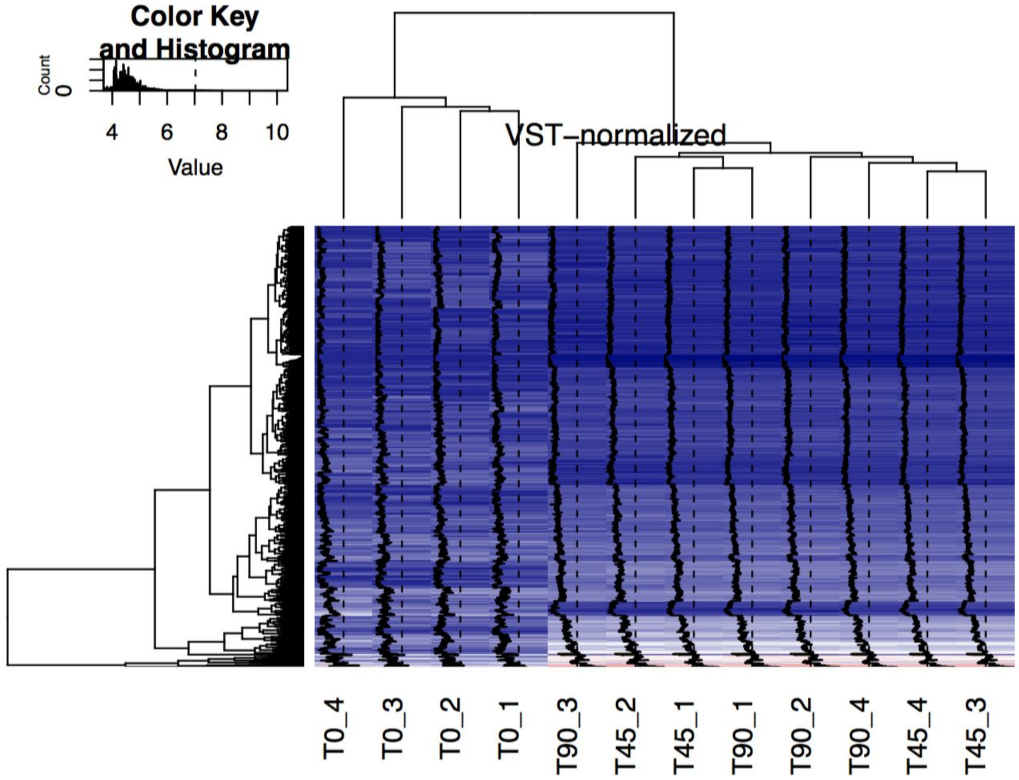

Fig. S10: Dataset clustering with VST-normalized Counts.

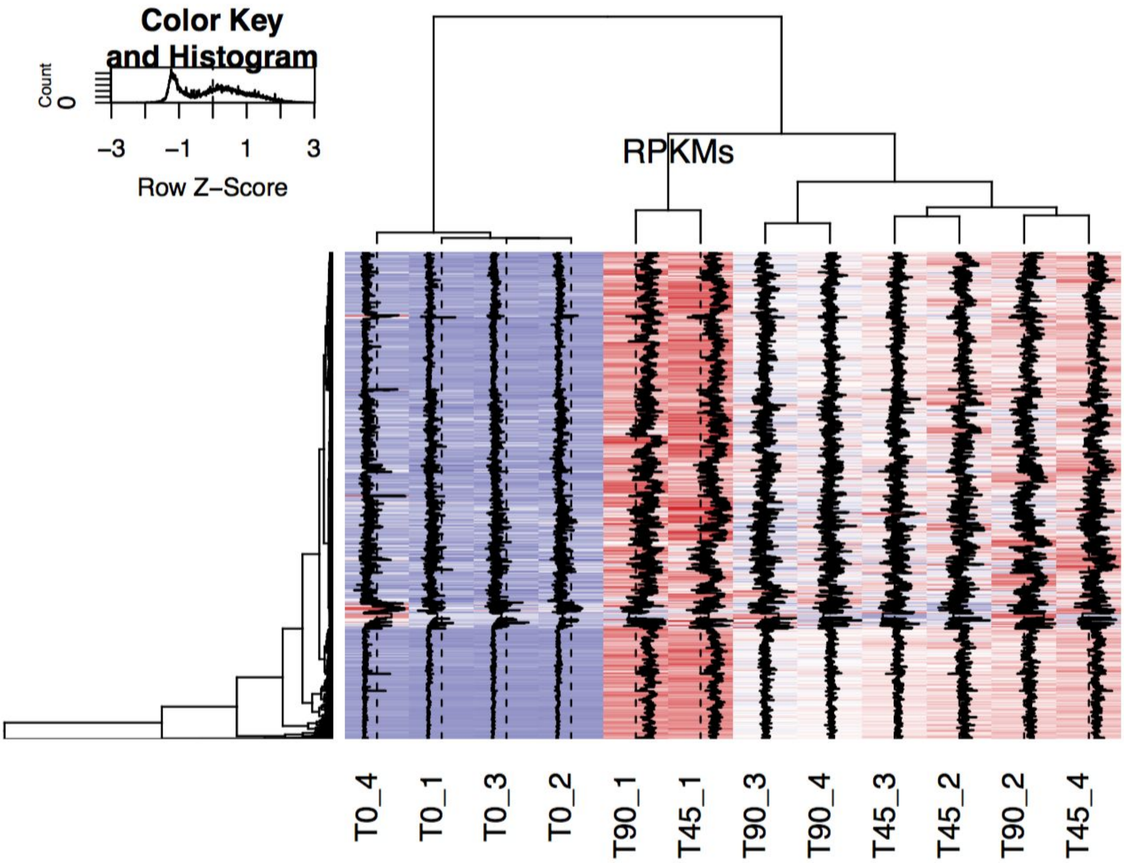

Fig. S11: Dataset clustering with RPKMs.

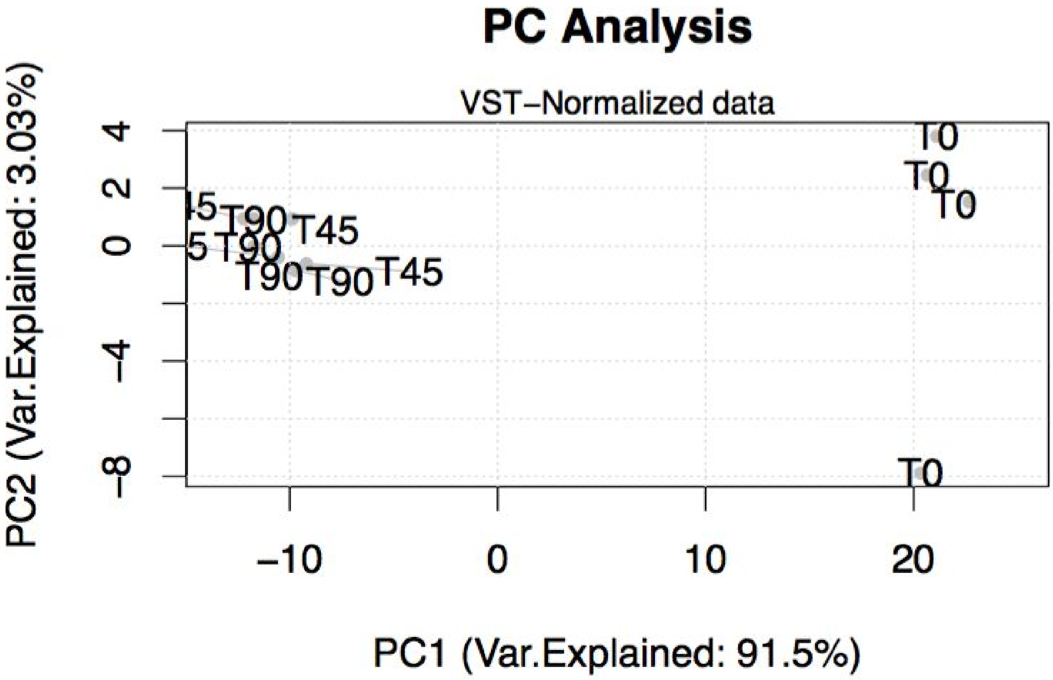

Fig. S12: Principal Component Analysis of the dataset, highlighting components 1 and 2.

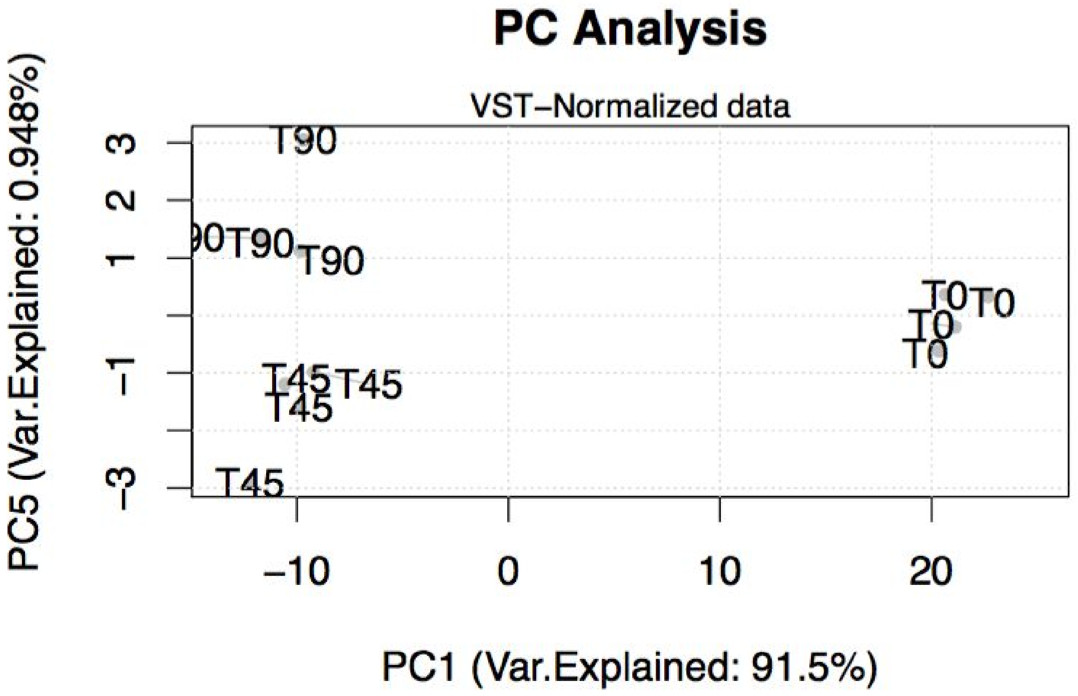

Fig. S13: Principal Component Analysis of the dataset, highlighting components 1 and 5.

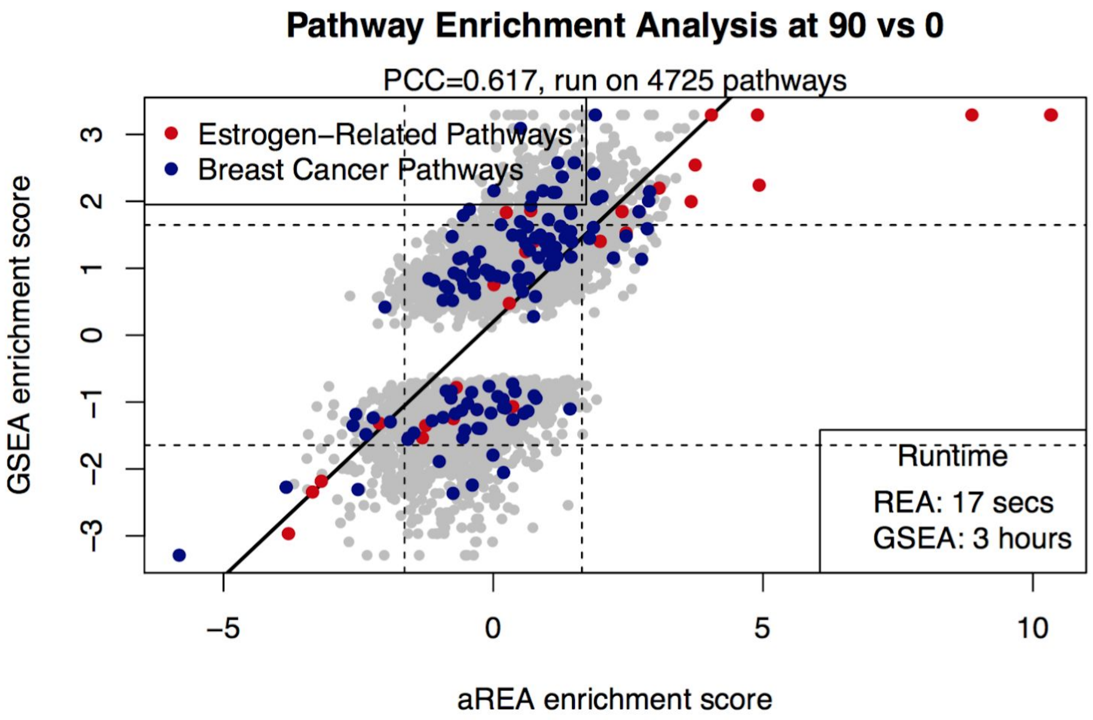

Fig. S14: Comparison of GSEA and aREA on a differential binding signature.

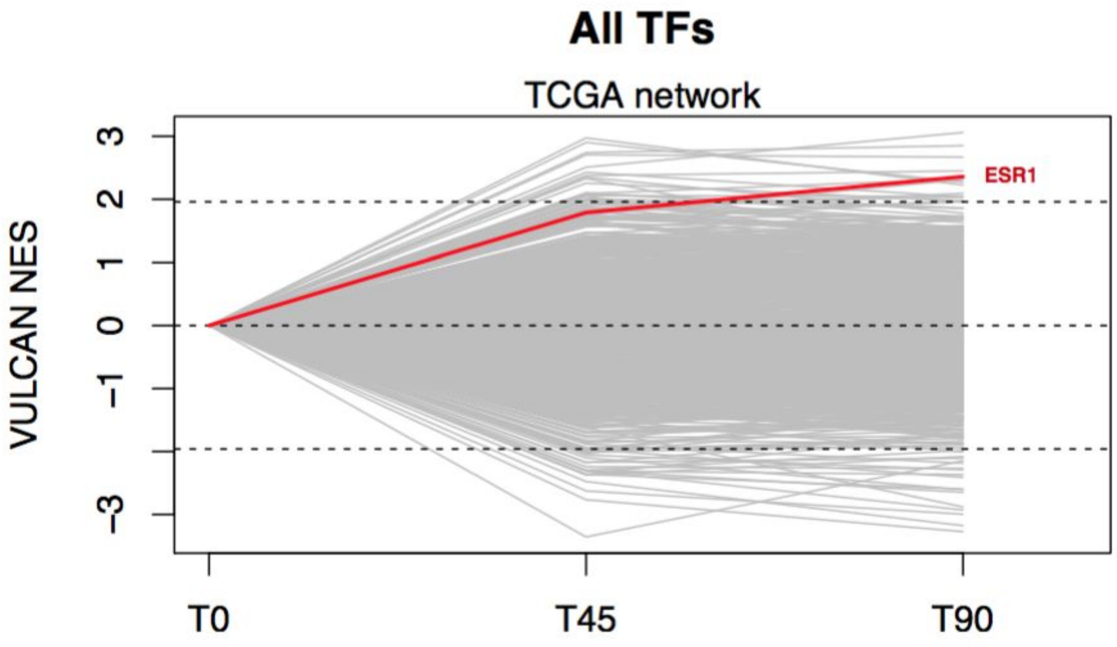

Fig. S15: Global TF activity after Estradiol Treatment in MCF7 cells, inferred using the TCGA network, highlighting the ESR1 TF as an example.

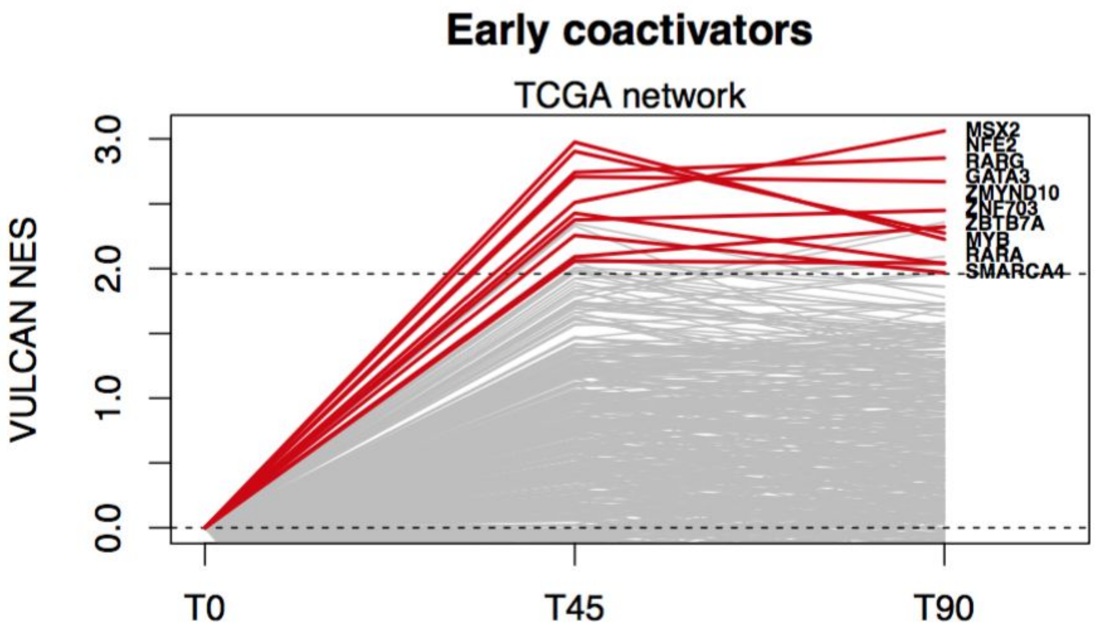

Fig. S16: Global TF activity after Estradiol Treatment in MCF7 cells, inferred using the TCGA network, highlighting TFs significantly upregulated at 45 minutes and 90 minutes.

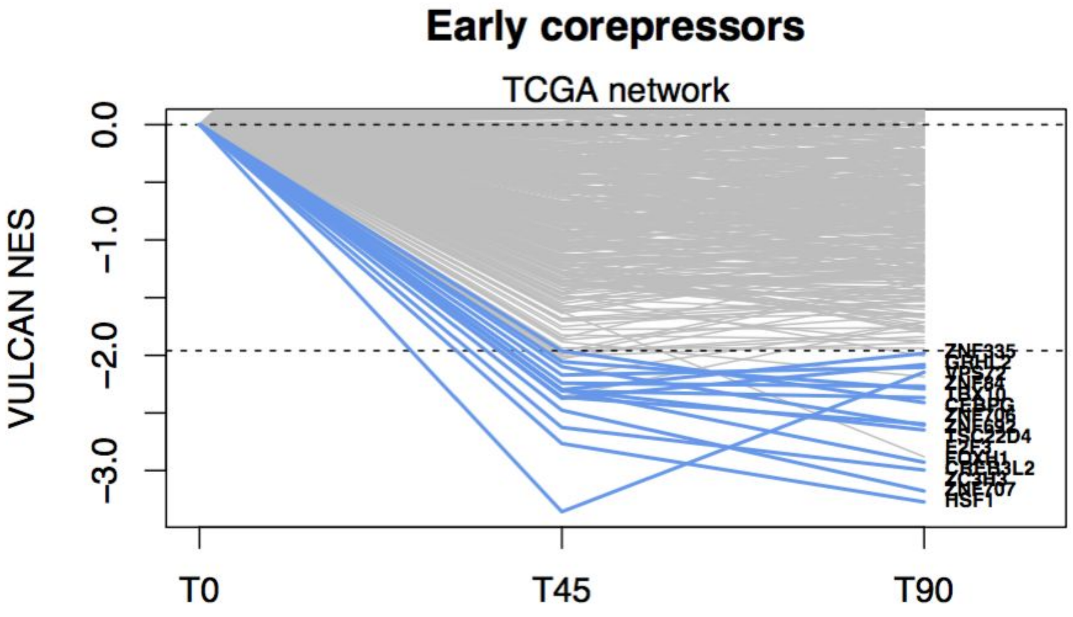

Fig. S17: Global TF activity after Estradiol Treatment in MCF7 cells, inferred using the TCGA network, highlighting TFs significantly downregulated at 45 minutes and 90 minutes.

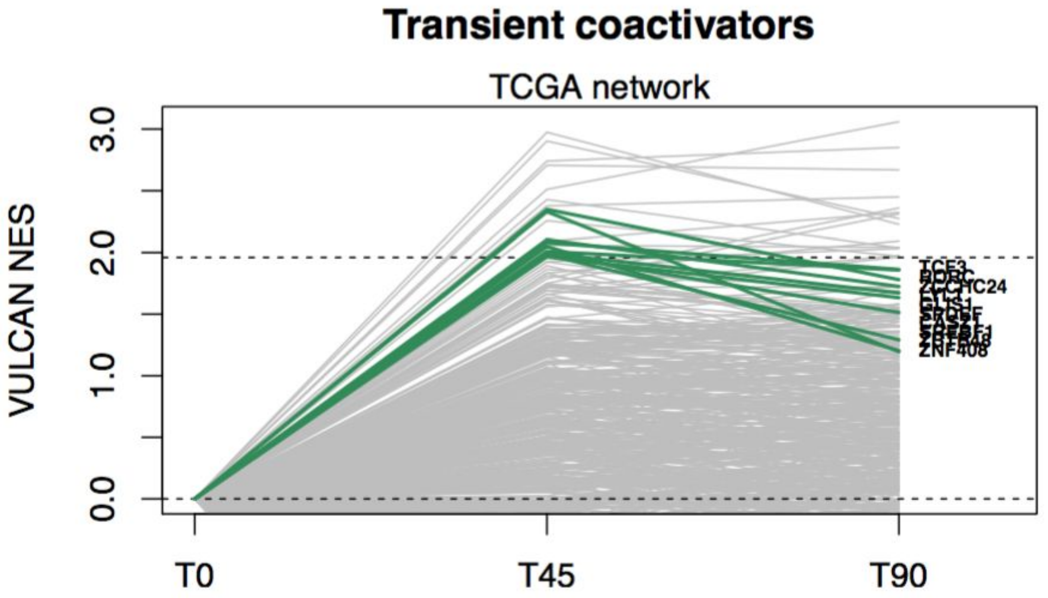

Fig. S18: Global TF activity after Estradiol Treatment in MCF7 cells, inferred using the TCGA network, highlighting TFs significantly upregulated at 45 minutes but not at 90 minutes.

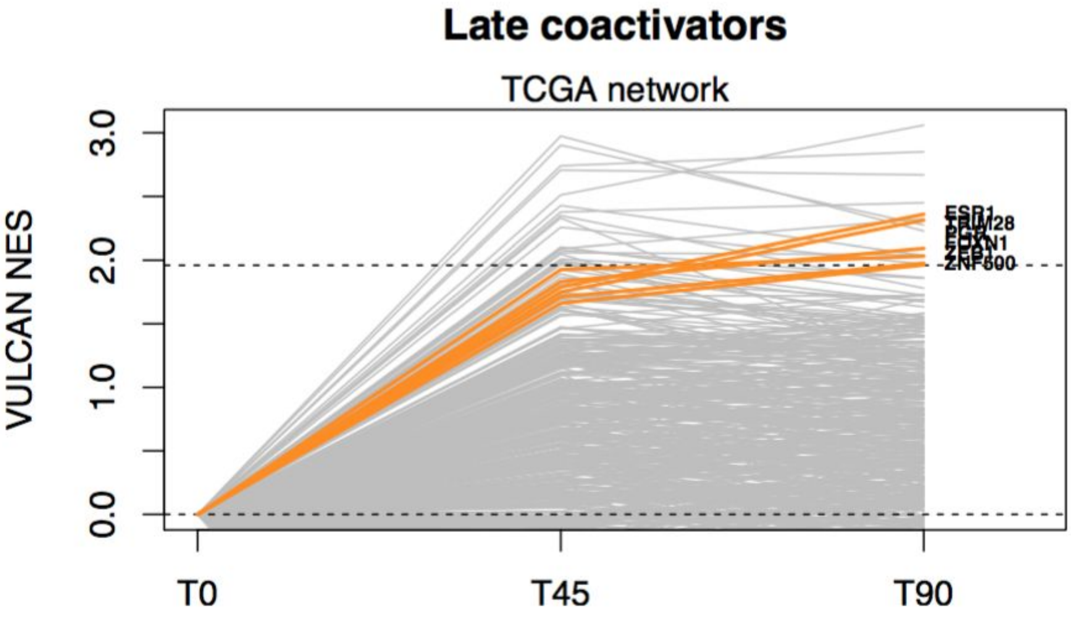

Fig. S19: Global TF activity after Estradiol Treatment in MCF7 cells, inferred using the TCGA network, highlighting TFs significantly upregulated at 90 minutes but not at 45 minutes.

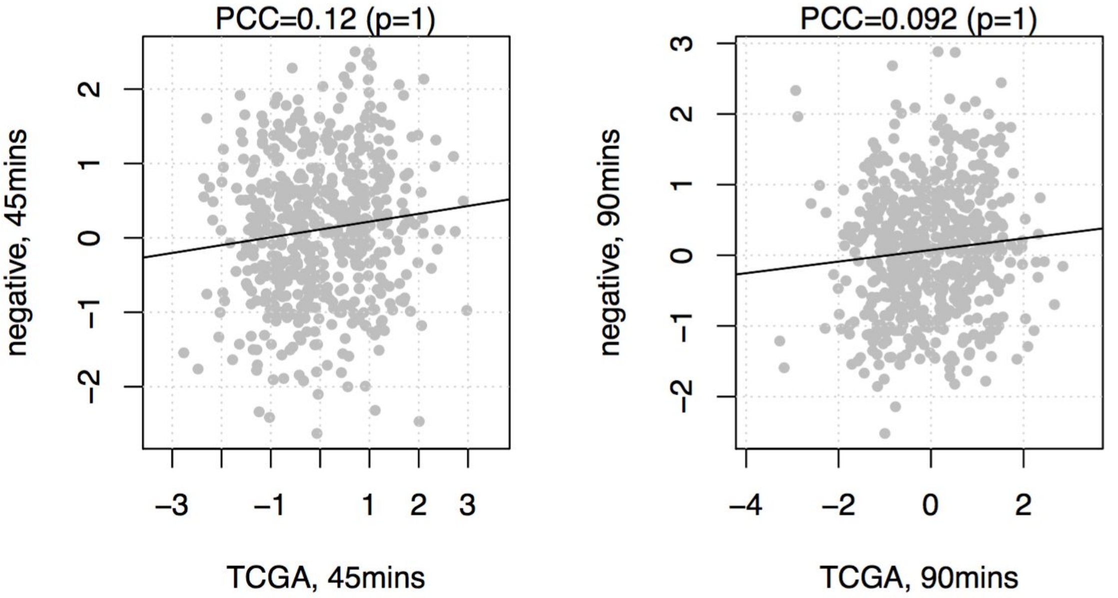

Fig. S20: Comparison between activities inferred through a breast cancer TCGA dataset and the AML dataset. PCC indicates the Pearson Correlation Coefficient.

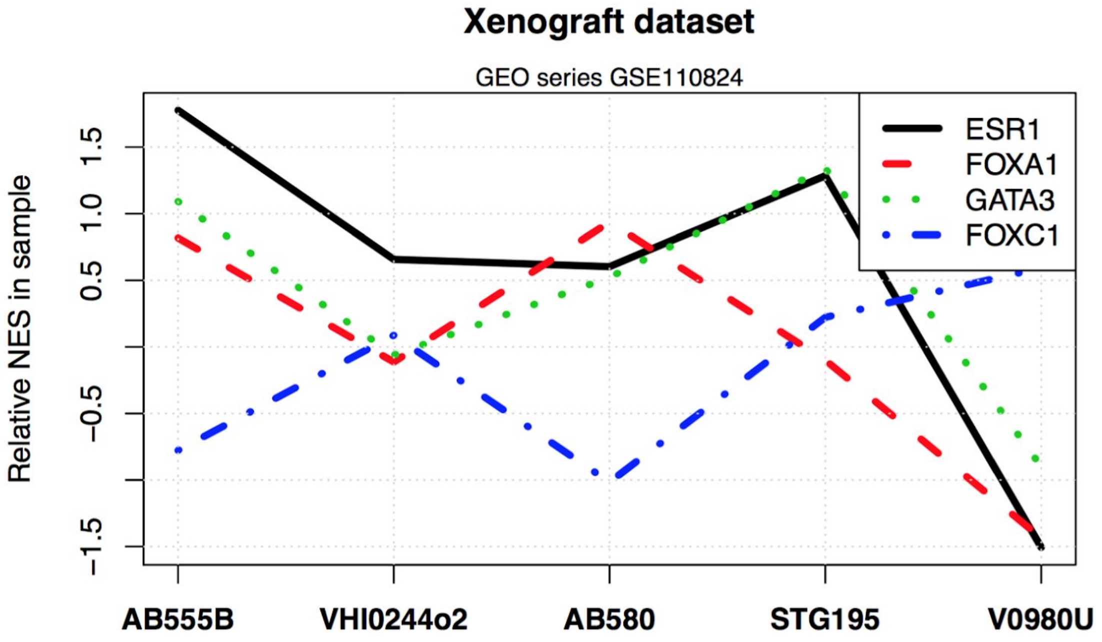

Fig. S21: VULCAN Activity scores for a few TFs extracted from the ER-targeted ChIP-Seq Xenograft (dataset GSE110824).

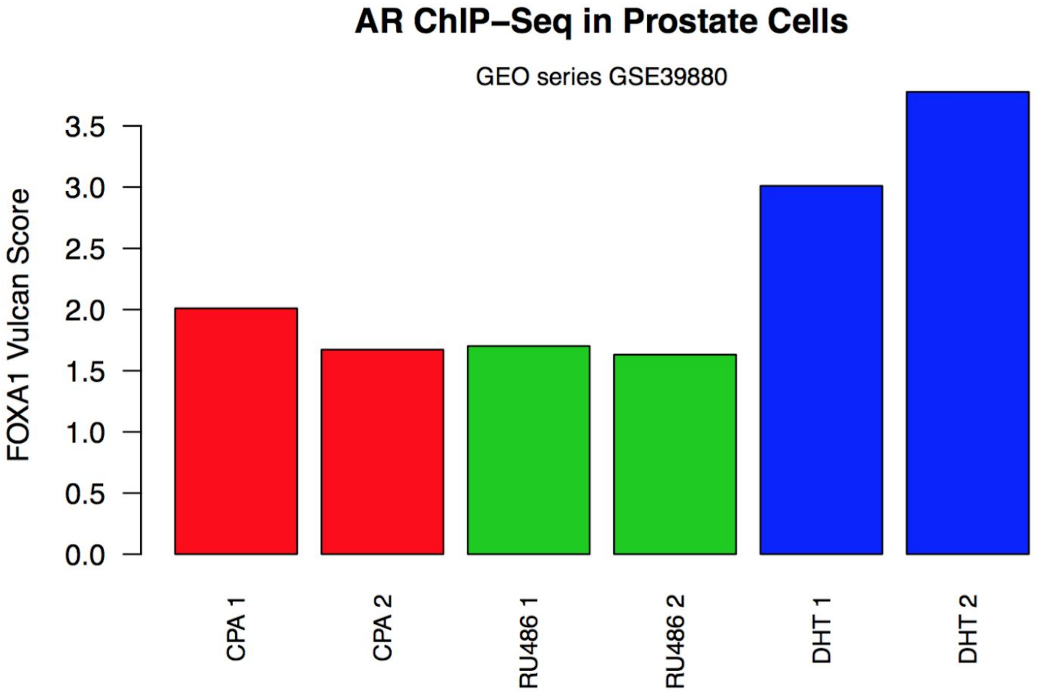

Fig. S22: VULCAN Activity scores for FOXA1 in Prostate cell lines (dataset GSE39880).

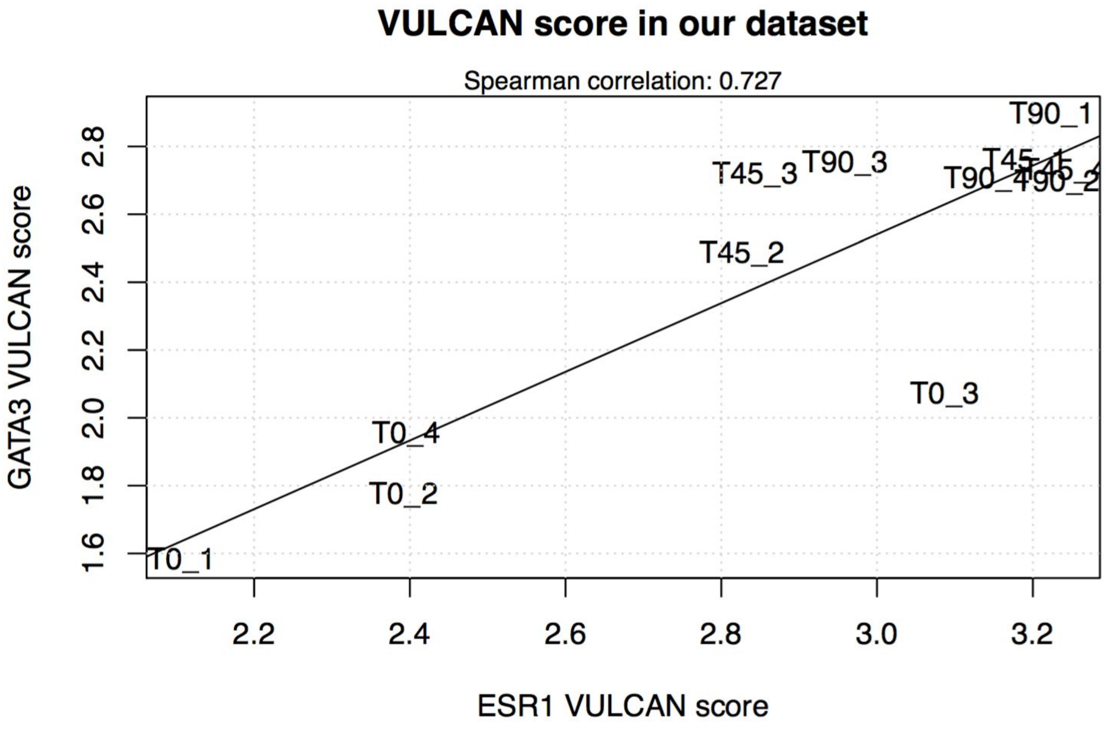

Fig. S23: VULCAN scores of GATA3 and ESR1 in our dataset. Individual samples are indicated.

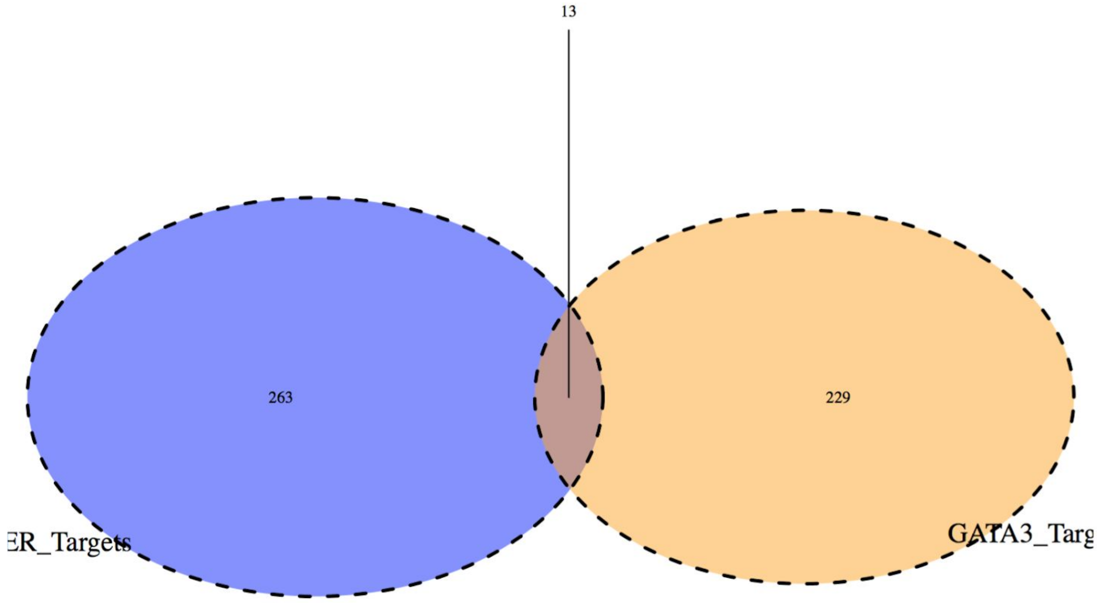

Fig. S24: Example of target intersection between GATA3 and ER according to the MSigDB database of canonical TF-specific motifs in putative target gene promoters.

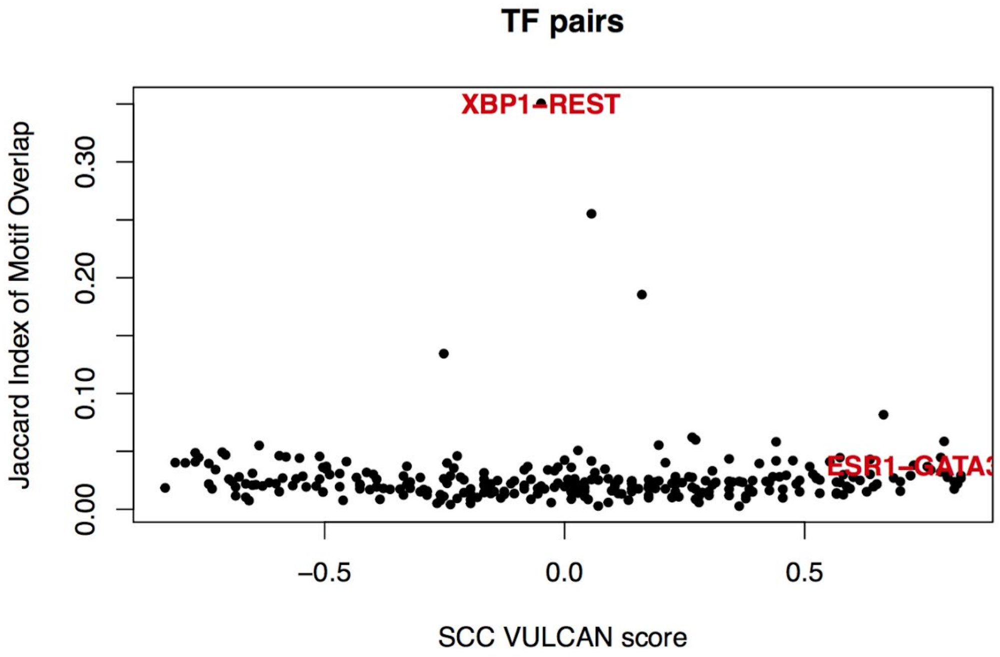

Fig. S25: TF pairs compared in terms of VULCAN score Spearman Correlation Coefficient in our ER dataset and in terms of Jaccard Index of motif-based target intersection according to the MSigDB C3 collection.

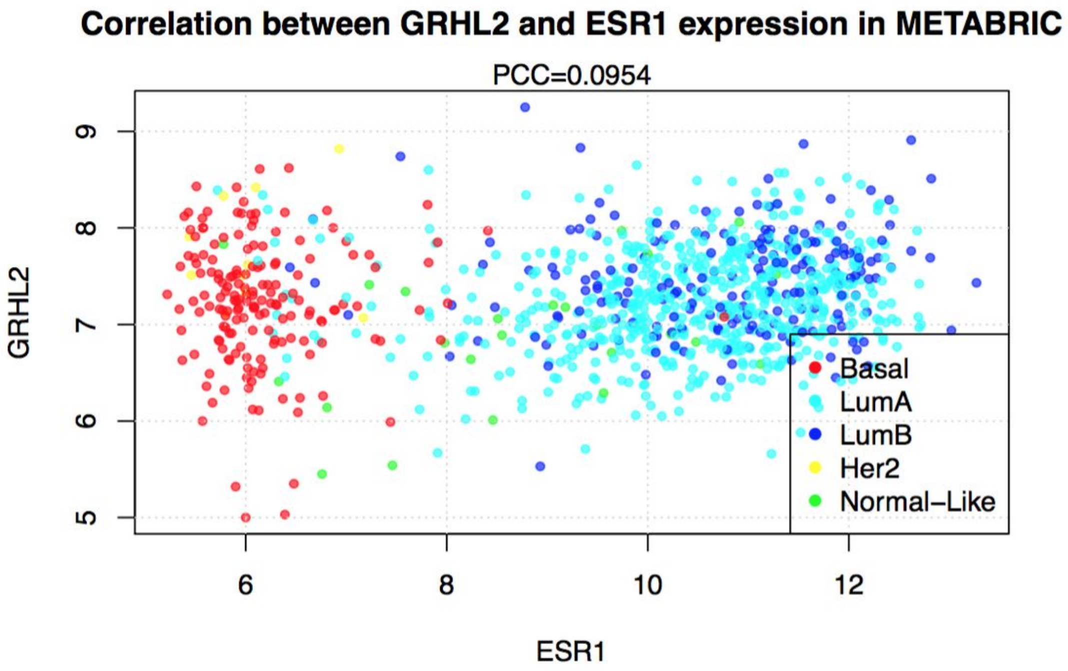

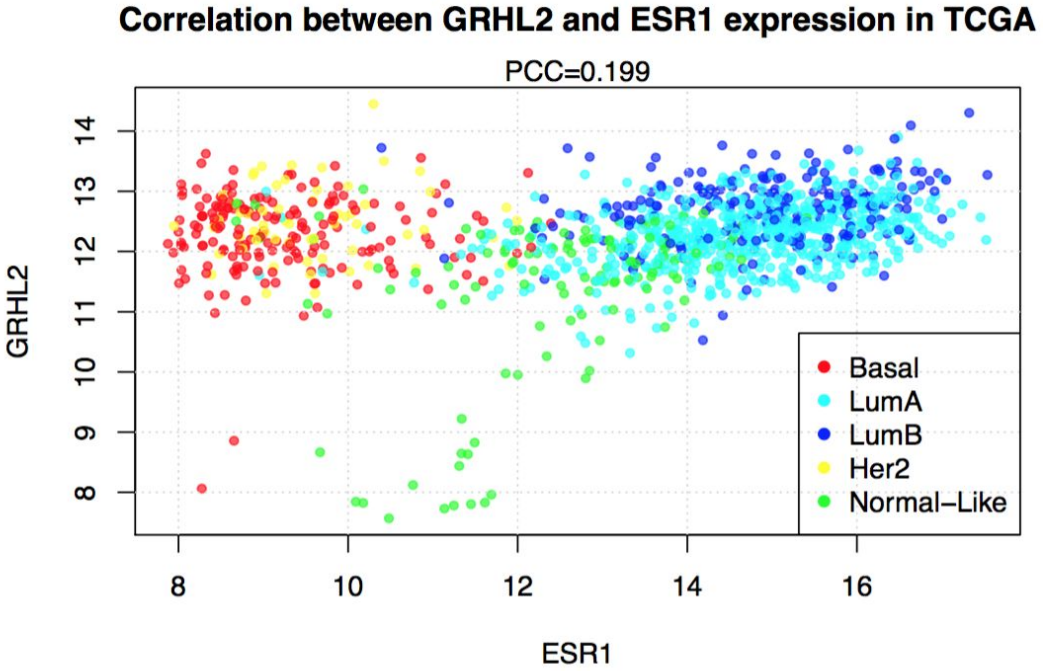

Fig. S26: Correlation between GRHL2 and ESR1 expression in the TCGA & METABRIC breast cancer datasets.

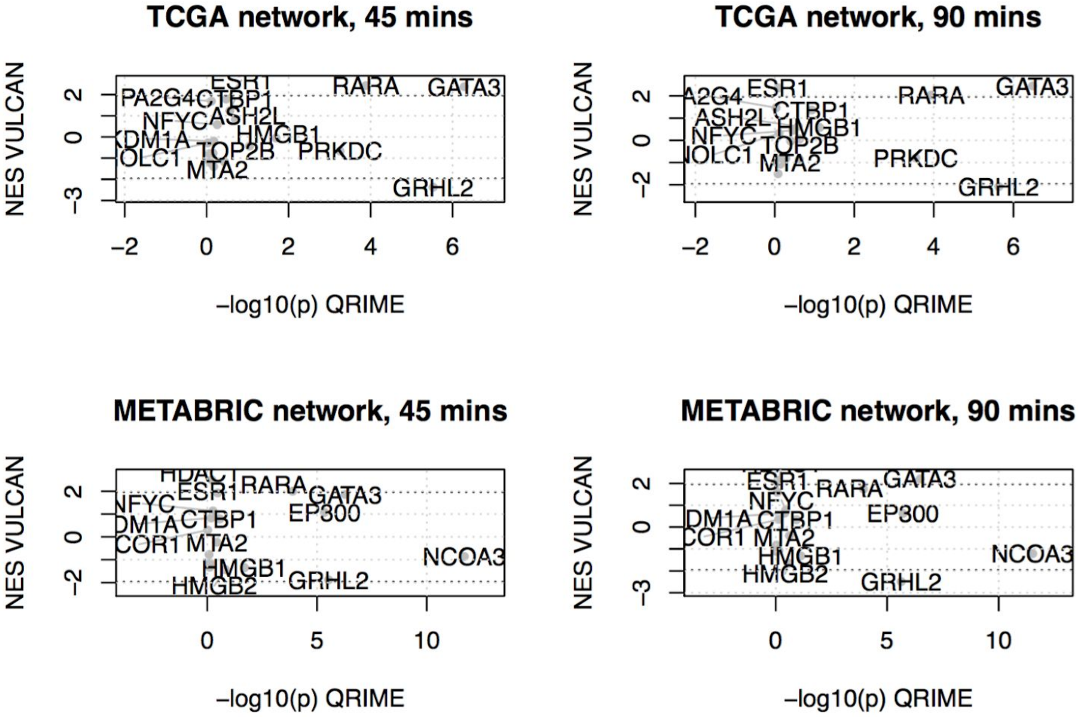

Fig. S27: Comparison of Normalized Enrichment Score between the QRIME method (x-axis) and the VULCAN method (y-axis) at two time points using two regulatory networks for VULCAN

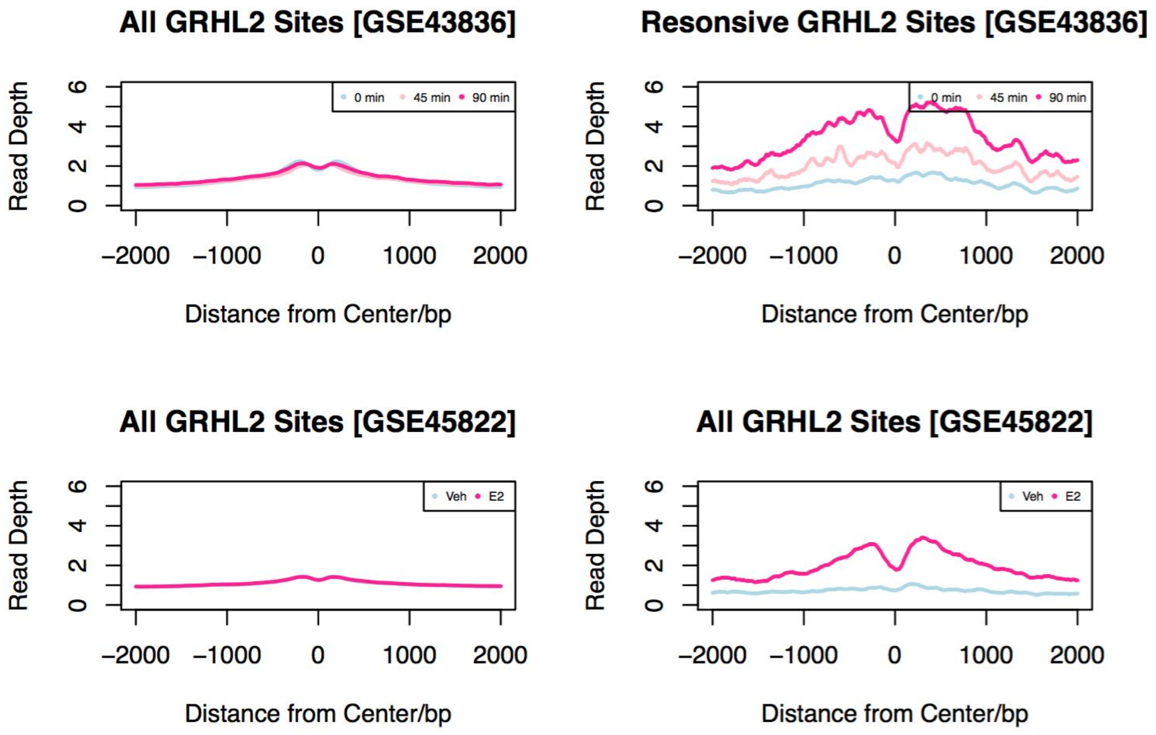

Fig. S28: Analysis of GRHL2 sites of Gro-Seq data from GSE43836 and GSE45822 both showed that E2 responsive GRHL2 responsive sites are transcriptionally responsive to E2.

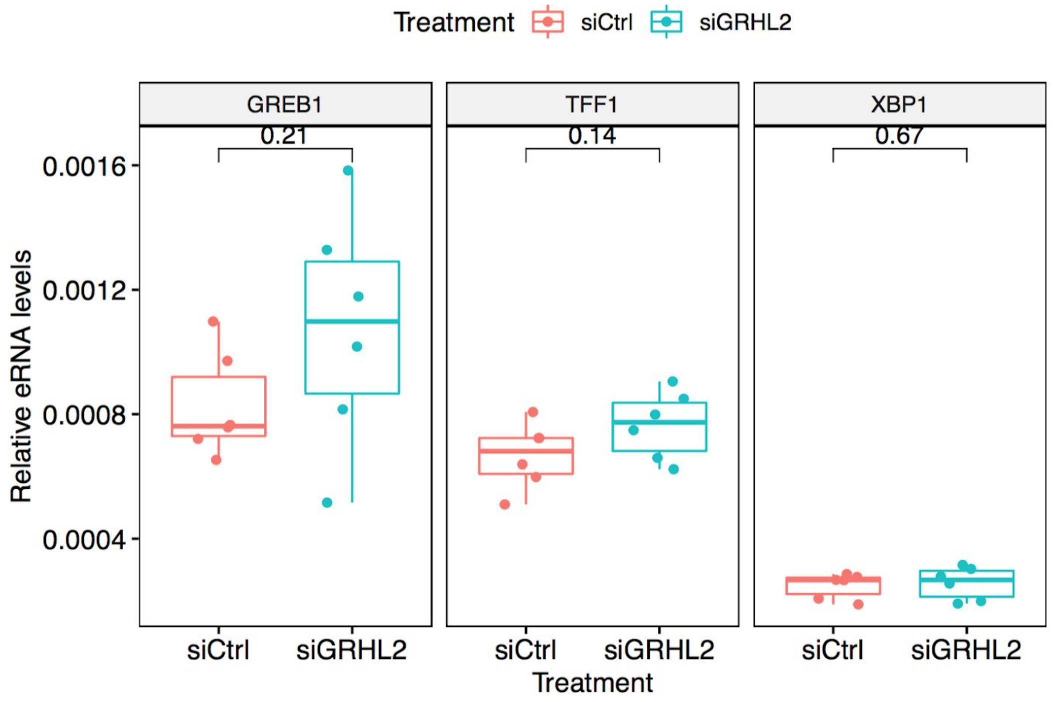

Fig. S29: Effect of knockdown of GRHL2 on eRNA at E2 responsive binding sites.

Supplementary Tables

	NES, 90' vs 0'	pvalue
Bhat esr1 targets not via akt1 up	10.3	4.6e-25
Bhat esr1 targets via akt1 up	10.3	5.3e-25
Dutertre estradiol response 6hr up	8.87	7.1e-19
Frasor response to estradiol up	4.93	8.3e-07
Dutertre estradiol response 24hr up	4.9	9.7e-07
Stein esr1 targets	4.04	5.3e-05
Stein esrra targets responsive to estrogen dn	3.74	0.00018
Creighton endocrine therapy resistance 1	3.72	2e-04
Stossi response to estradiol	3.67	0.00024
Zwang egf interval dn	3.55	0.00039
Creighton endocrine therapy resistance 4	3.42	0.00062
Pedrioli mir31 targets up	3.38	0.00071
Massarweh tamoxifen resistance dn	3.24	0.0012
Lein pons markers	3.19	0.0014
Reactome hs gag degradation	3.19	0.0014
Jiang tip30 targets dn	3.17	0.0015
Kegg glycerophospholipid metabolism	3.16	0.0016
Geserick tert targets dn	3.13	0.0018
Gross hypoxia via hif1a dn	3.09	0.002
Valk aml cluster 6	3.09	0.002

Table S1: aREA results: upregulated MsigDB pathways at 90mins.

	NES, 45' vs 0'	pvalue
Bhat esr1 targets via akt1 up	10.8	3e-27
Bhat esr1 targets not via akt1 up	10.4	2.8e-25
Dutertre estradiol response 6hr up	8.1	5.5e-16
Frasor response to estradiol up	4.54	5.6e-06
Stein esrra targets responsive to estrogen dn	4.21	2.6e-05
Dutertre estradiol response 24hr up	4.15	3.4e-05
Stein esr1 targets	4.03	5.6e-05
Vantveer breast cancer esr1 up	3.84	0.00012
Geserick tert targets dn	3.69	0.00022
Pedrioli mir31 targets up	3.61	3e-04
Reactome glycerophospholipid biosynthesis	3.39	0.00069
Zwang egf interval dn	3.38	0.00072
Naba ecm affiliated	3.31	0.00093
Stossi response to estradiol	3.29	0.00099
Lien breast carcinoma metaplastic vs ductal dn	3.25	0.0011
Kegg glycerophospholipid metabolism	3.25	0.0012
Creighton endocrine therapy resistance 1	3.24	0.0012
Reactome hs gag degradation	3.21	0.0013
Valk aml cluster 6	3.2	0.0014
Massarweh tamoxifen resistance dn	3.1	0.0019

Table S2: aREA results: upregulated MsigDBpathways at 45mins.
